# Retroperitoneal Teratoma in Newborn Mimicking Neuroblastoma With Rapid Growth

**DOI:** 10.1155/crpe/9630009

**Published:** 2026-06-17

**Authors:** Reina Murasawa, Daisuke Watanabe, Nobuyuki Katsumata, Hiroko Oshiro, Satoshi Shinohara, Rei Sunami, Tamami Fukatsu, Norio Hasuda, Yuka Yokota, Yukihiro Sugita, Koshi Akahane, Kumiko Goi, Atsushi Nemoto, Takeshi Inukai, Atsushi Naitoh

**Affiliations:** ^1^ Department of Neonatology, Perinatal Center, Yamanashi Prefectural Central Hospital, Kofu, Yamanashi, Japan, ych.pref.yamanashi.jp; ^2^ Department of Pediatrics, Faculty of Medicine, University of Yamanashi, Chuo, Yamanashi, Japan, yamanashi.ac.jp; ^3^ Department of Obstetrics and Gynecology, Perinatal Center, Yamanashi Prefectural Central Hospital, Kofu, Yamanashi, Japan, ych.pref.yamanashi.jp; ^4^ Department of Pediatric Surgery, Faculty of Medicine, University of Yamanashi, Chuo, Yamanashi, Japan, yamanashi.ac.jp; ^5^ Department of Pathology, Faculty of Medicine, University of Yamanashi, Chuo, Yamanashi, Japan, yamanashi.ac.jp

**Keywords:** neonate, neuroblastoma, rapid growth, retroperitoneal teratoma

## Abstract

Adrenal‐region masses during the perinatal period are uncommon and may include neuroblastoma and teratoma. These two entities often show overlapping features on initial imaging. Differentiation is challenging because teratomas may initially lack visible fat or coarse calcification, whereas congenital neuroblastoma may present with normal catecholamine levels and no abnormal ^123^I‐MIBG uptake. In the present patient, a rapidly enlarging right suprarenal mass was identified during late gestation. The mass was initially interpreted as neuroblastoma based on its cystic–solid appearance and nonspecific biochemical findings. After birth, the mass continued to grow, and the initial postnatal evaluation was inconclusive, showing no abnormal MIBG uptake and normal catecholamine metabolites. However, serial imaging revealed time‐dependent changes, including new calcification, emerging fatty components, and progressive enlargement, prompting reconsideration of the initial diagnosis. Complete resection confirmed an immature retroperitoneal teratoma without adrenal involvement. These observations illustrate that a retroperitoneal teratoma can closely mimic an adrenal neuroblastoma during the perinatal period, particularly during the fetal and early neonatal periods, when fat or calcification is not yet apparent. Time‐dependent radiologic evolution is therefore a key diagnostic clue, and continuous fetal‐to‐neonatal imaging follow‐up plays a crucial role in accurately distinguishing these tumors.

## 1. Introduction

Teratomas are germ cell tumors composed of tissues derived from all three germ layers: ectoderm, mesoderm, and endoderm [1]. Among tumors detected in late gestation or early neonatal life, teratomas are among the most common, with an estimated incidence of 1 in 20,000–40,000 live births [2, 3]. The sacrococcygeal region is the most common primary site, accounting for nearly 40% of cases, whereas retroperitoneal or adrenal teratomas are rare, accounting for 2%–5% [1, 4].

Adrenal‐region masses identified in the perinatal period encompass a broad range of conditions, including neuroblastoma—the most common malignant tumor in this age group—as well as adrenal hemorrhage, pulmonary sequestration, enteric duplication cysts, adrenal cytomegaly, and teratoma [2, 5, 6]. In older infants and children, neuroblastoma and teratoma can often be differentiated using radiologic and biochemical features. Neuroblastomas typically show ^123^I‐MIBG uptake and elevated urinary catecholamine metabolites (VMA and HVA) [5, 6], whereas teratomas more often show macroscopic fat or coarse calcification and may be associated with elevated AFP or *β*‐hCG [1].

However, in the perinatal period, early static evaluation may be nondiagnostic because diagnostic components can emerge over time. Several studies have reported perinatal teratomas that were initially presumed to be adrenal‐origin neuroblastomas [2, 7–12]. We report a perinatal retroperitoneal teratoma that initially mimicked an adrenal neuroblastoma, highlighting the importance of continuous fetal‐to‐neonatal imaging follow‐up when early evaluation of an adrenal‐region mass is nondiagnostic. This case also illustrates that reliance on a single early imaging time point may be insufficient, as interval imaging can reveal diagnostic features that become apparent during postnatal growth.

## 2. Case Presentation

**FIGURE 1:**
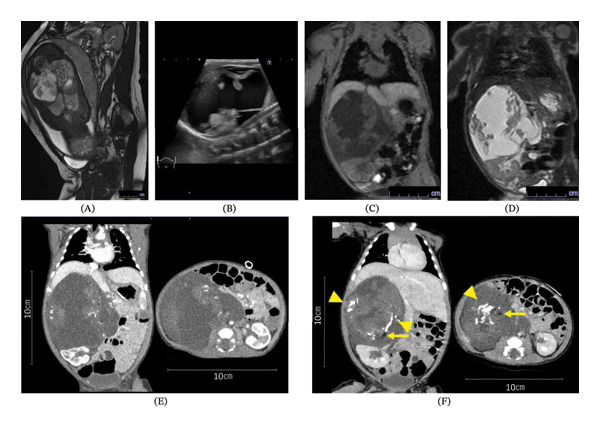
Imaging findings. (A) Fetal MRI at 36 weeks’ gestation demonstrates a 58‐mm cystic–solid mass located between the right kidney and liver. The lesion is predominantly cystic with a small solid component. (B) Postnatal ultrasonography on Day 5 shows an 80‐mm cystic–solid mass in the same region; the right adrenal gland is not clearly identified. (C) Postnatal T1‐weighted MRI on Day 5 reveals a 77‐mm cystic–solid mass at the upper pole of the right kidney; the solid component is isointense. (D) Postnatal T2‐weighted MRI on Day 5 shows that the solid component is hypointense. (E) Contrast‐enhanced CT on Day 7 demonstrates an 80 × 78‐mm cystic–solid mass with coarse calcifications; the right adrenal gland is not identified. (F) CT on Day 23 shows newly apparent fatty components (arrow) and linear calcifications (arrowhead) within the mass, which measures 84 mm in maximum diameter.

A 34‐year‐old primigravida conceived naturally and received routine antenatal care at a local clinic. At 34 weeks of gestation, fetal ultrasonography revealed a 30‐mm mass located superior to the right kidney. At 35 weeks, the mass enlarged to 36 mm, and the right adrenal gland was not visualized. Fetal magnetic resonance imaging (MRI) at 36 weeks demonstrated a 58‐mm cystic mass with internal solid components, raising suspicion for neuroblastoma (Figure 1A).

At 38 + 1 weeks of gestation, the mother underwent an emergency cesarean section due to arrest of labor. The female neonate weighed 3146 g, and Apgar scores were 8 and 9 at 1 and 5 min, respectively. Vomiting occurred shortly after birth. Gastrointestinal compression caused by the abdominal mass was suspected. Oral intake was restricted, and a nasogastric tube was inserted to control vomiting. Laboratory studies did not support neuroblastoma (NSE 28.1 ng/mL; urinary VMA 7.6 μg/mg creatinine; HVA 19.6 μg/mg creatinine). No evidence of malignancy was observed, including elevated LDH levels or blast cells. CRP was also negative. AFP was markedly elevated (105,133.2 ng/mL) but remained within the age‐specific reference range. ^123^I‐MIBG scintigraphy showed no abnormal uptake. Postnatal imaging on Day 5, including ultrasonography (Figure 1B) and MRI (Figure 1C,D), demonstrated a 77‐mm cystic mass with septations and irregular nodules; the right adrenal gland remained unidentifiable.

**FIGURE 2:**
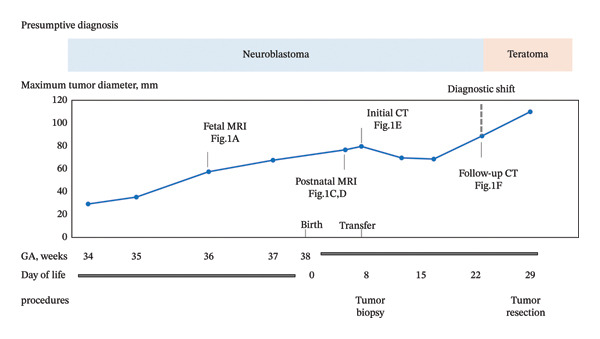
Timeline of tumor size, diagnostic evaluations, and provisional diagnoses. The line graph shows changes in tumor size (maximum diameter) from the fetal period to surgical resection. Symbols indicate the timing of imaging examinations, biopsy, and surgery, including postnatal MRI on Day 5. Horizontal bars represent provisional clinical diagnoses assigned at each time point based on available imaging and laboratory findings. GA, gestational age; MRI, magnetic resonance imaging; CT, computed tomography.

The neonate was transferred to a tertiary care center on Day 7. Computed tomography (CT) revealed an 80‐mm mixed cystic and solid mass containing calcifications (Figure 1E). An incisional biopsy was performed on Day 8. A transverse incision was made in the right abdomen. After aspiration of 59 mL of fluid from the mass, tissue samples were obtained from the tumor wall and solid components. The biopsy revealed mature adipose tissue and intestinal‐type epithelium with goblet cells. Only a few synaptophysin‐positive neuroectodermal cells were observed, making neuroblastoma unlikely. Serial ultrasonography revealed continued tumor enlargement. The biopsy did not establish a definitive diagnosis, so total excision was planned. Preoperative CT on Day 23 demonstrated more conspicuous macroscopic fat and calcification, features suggestive of a teratoma (Figure 1F). Total tumor resection was performed on Day 28 through a right subcostal oblique incision. The tumor was separated from the surrounding organs and resected from the retroperitoneal space. The tumor measured 110 × 60 × 55 mm. The right adrenal gland, which had been compressed between the tumor and the liver, was also resected because tumor involvement could not be ruled out intraoperatively. Grossly, the resected tumor consisted of mixed cystic and solid components, including fatty areas and calcified structures. Histologically, the tumor consisted of a mixture of various tissues, including epidermis, central nervous system tissue, fat, bone, cartilage, and teeth. These findings were consistent with the fatty and calcified components observed on the preoperative CT scan. Additionally, immature neuroepithelial components with neural tube structures and rosette formations were identified. The additional adrenal specimen showed no neoplastic lesions, indicating that the tumor originated in the retroperitoneum. Based on these findings, the preoperative diagnosis of teratoma was supported, and the histological diagnosis was a Grade 3 immature teratoma (Norris classification). The tumor was classified as Stage II (Children’s Oncology Group surgical staging system). The postoperative course was uneventful, and no recurrence was observed at the 1‐year follow‐up. The clinical course from the fetal period to tumor resection is summarized in Figure 2.

## 3. Discussion

In this case, early evaluation of an adrenal‐region mass remained nondiagnostic, whereas continuous fetal‐to‐neonatal imaging follow‐up revealed increasingly conspicuous macroscopic fat and calcification during postnatal growth. These time‐dependent changes prompted revision of the working diagnosis from suspected adrenal neuroblastoma to retroperitoneal teratoma before definitive resection.

This diagnostic trajectory is consistent with prior perinatal reports in which retroperitoneal or adrenal teratomas were initially interpreted as neuroblastomas (Tables 1 and 2) [2, 7–12]. Early fetal or immediate postnatal imaging often shows mixed cystic–solid architecture with absent or subtle fat and calcification, and tumor size can obscure the true anatomical origin (Table 1). In addition, tumor markers reported in these cases were largely unremarkable and did not contribute to definitive differentiation at the initial evaluation (Table 2). A similar diagnostic challenge involving another adrenal‐region differential diagnosis was described by Yasui et al., who reported a mass initially suspected to represent adrenal hemorrhage but later diagnosed preoperatively as a teratoma after interval imaging showed a change from a cystic lesion at birth to a predominantly solid mass with fatty and calcified components by 4 months of age [13]. Accordingly, reliance on a single early imaging time point may be insufficient to distinguish teratoma from other perinatal adrenal‐region masses.

**TABLE 1:** Patient characteristics, serial imaging findings, diagnostic course, and clinical outcomes of the present case and previously reported cases.

Patient no.	Our patient	P1	P2	P3	P4	P5	P6	P7	P8
Reference		Yi CM, et al. (2005) [7]	Asai S, et al. (2007) [2]	Maddali R, et al. (2024) [10]	Poyyamozhy KI, et al. (2024) [12]	Yasui T, et al. (2013) [13]	Garg R, et al. (2015) [8]	Garcia C, et al. (2023) [9]	Msarweh A, et al. (2024) [11]
GA at delivery (weeks)	38	39	39	39	Term	Term	Term	NR	NR
Sex	F	F	F	F	F	M	F	M	F
GA at detection (weeks)	34	38	33	31	NR	37	32	22	30
Initial diagnosis	NB	NB	NB	NB^∗^	NB^∗∗^	NB^∗∗∗^	NB	NB	NB
Tumor size (mm)	110	130	80	86	120	21	76	22	90
CT	Mixed	Mixed	Mixed	Mixed	Mixed	Cystic (MRI, no early CT)	Cystic	Mixed	Mixed
Calcification	+ Linear	+ Teeth‐like	+ Linear	+	+	—	—	—	+
Fat	−⟶+	+	NR	—	NR	—	—	NR	+
Repeat postnatal CT	Yes	No	No	No	No	No	No	No	No
Follow‐up imaging	Calcification pattern change fat appeared	Calcification pattern change	Calcification pattern change	NR	NR	Cystic to solid change calcification and fat appeared	NR	No change	NR
Biopsy	+ (Nondiagnostic)	—	—	—	—	—	+ (Nondiagnostic)	—	—
Final diagnosis	IMT	IMT	IMT	IMT	MT	MT	MT	MT	MT
Outcome	Alive (1y)	NR	Alive (1y)	NR	Alive (1y)	NR	Alive (6m)	Alive (1y5m)	Alive (1y)

*Note:* NB: neuroblastoma, PreN: prenatal, US: ultrasound, calc: calcification, IMT: immature teratoma, and repeat postnatal CT: CT performed at ≥ 2 postnatal time points (to assess evolution). Follow‐up imaging includes prenatal US and postnatal imaging when reported.

Abbreviations: DOL: day of life; GA, gestational age; m/y, months/year; MT, mature teratoma; NR, not reported.

^∗^Differentials considered teratoma and adrenal hemorrhage.

^∗∗^Differentials considered teratoma and lymphangioma.

^∗∗∗^Differentials considered adrenal hemorrhage.

**TABLE 2:** Tumor markers and MIBG scintigraphy in the present case and previously reported cases.

Patient no.	Our patient	P1	P2	P3	P4	P5	P6	P7	P8
Reference		Yi CM, et al. (2005) [7]	Asai S, et al. (2007) [2]	Maddali R, et al. (2024) [10]	Poyyamozhy KI, et al. (2024) [12]	Yasui T, et al. (2013) [13]	Garg R, et al. (2015) [8]	Garcia C, et al. (2023) [9]	Msarweh A, et al. (2024) [11]
VMA (μg/mg creatinine)	Normal (7.6)	Normal (NR value)	NR	NR	NR	Normal (NR value)	Normal (NR value)	NR	Normal (NR value)
HVA (μg/mg creatinine)	Normal (19.6)	Normal (NR value)	NR	Normal (8.3)	NR	Normal (NR value)	Normal (NR value)	NR	Normal (NR value)
AFP (ng/mL)	Normal (105,133)	NR	Normal (62,200)	Normal (95,343)	Normal (NR value)	Normal (NR value)	NR	NR	Normal (NR value)
β‐hCG (mIU/mL)	Normal (2.4)	NR	Normal (0.07)	Normal (< 6)	Normal (NR value)	Normal (NR value)	NR	NR	Normal (NR value)
NSE (ng/mL)	Mildly elevated (28.1)	NR	NR	NR	NR	NR	NR	NR	NR
MIBG scan	Normal	Normal	NR	Normal	NR	Normal	NR	NR	NR

*Note:* Reference ranges (our institution): VMA 8.6 ± 4.1 μg/mg creatinine; HVA 18.1 ± 6.18 μg/mg creatinine; NSE ≦ 15 ng/mL; *β*‐hCG < 50 mIU/mL. AFP is age‐dependent, interpreted using age‐specific ranges.

Large retroperitoneal tumors may displace and compress the adrenal gland, creating an adrenal‐origin impression when the gland is not visualized on fetal or early postnatal imaging. In our patient, the right adrenal gland was not identifiable on early studies, which biased interpretation toward an adrenal lesion; operative findings later confirmed adrenal displacement without invasion, supporting a retroperitoneal origin. Similar “adrenal mimicry” has been described in adults with large retroperitoneal tumors misinterpreted as adrenal lesions [14]; however, extrapolation to neonates should be made cautiously because neonatal anatomy and imaging constraints differ.

Neuroblastoma‐oriented tests may also be noncontributory in congenital or neonatal presentations. While ^123^I‐MIBG scintigraphy and urinary catecholamine metabolites are useful in many neuroblastomas, negative results should be interpreted in conjunction with the clinical course and longitudinal imaging rather than in isolation [5]. In addition, AFP is physiologically high in neonates; therefore, age‐specific reference ranges are essential and may limit the discriminatory value early in life. Collectively, when early workup is inconclusive, reassessment based on interval imaging—particularly the emergence of macroscopic fat and calcification—may help refine the diagnosis prior to surgery. Accurate preoperative characterization can help avoid unnecessary chemotherapy for teratomas and support surgical planning toward complete resection. It can also help avoid overly aggressive initial surgery for neuroblastoma, in which observation or neoadjuvant therapy may be preferable depending on risk and resectability.

Serial imaging may be informative in evaluating perinatal adrenal‐region tumors because diagnostic clues such as emerging calcification, macroscopic fat, and interval tumor growth can become apparent only over time. Although standardized follow‐up intervals have not been established, the frequency of follow‐up examinations should be individualized according to tumor size, symptoms, growth rate, and changes in imaging characteristics. Ultrasonography is useful as a first‐line modality for serial assessment because it can evaluate tumor size and internal characteristics without radiation exposure. In larger, symptomatic, or rapidly enlarging tumors, short‐interval follow‐up may be considered during the early neonatal period. For smaller and stable lesions, longer intervals may be reasonable. If changes in tumor characteristics are observed, or if the anatomical origin or relationship to adjacent organs remains unclear, MRI should be considered. CT may also be considered when calcification must be assessed for diagnosis or surgical planning, with careful attention to radiation exposure. In the present patient, early imaging lacked calcification, whereas subsequent studies demonstrated macroscopic fat, new calcification, and rapid enlargement of the mass, prompting diagnostic revision before surgery. Consistent with prior neonatal reports, cases without longitudinal imaging (P3, P4, P6, and P8) often remained indeterminate until resection. In contrast, cases with repeated imaging, including prenatal ultrasonography and postnatal CT (P1 and P2), documented progressive changes that supported diagnostic revision. These findings suggest that reassessment with serial imaging may provide additional diagnostic clues when the initial workup is inconclusive or tissue confirmation is not feasible.

## 4. Conclusion

In conclusion, this case illustrates that when early evaluation of a perinatal adrenal‐region mass is nondiagnostic, interval imaging can reveal evolving macroscopic fat and calcification that supports preoperative revision from suspected adrenal neuroblastoma to retroperitoneal teratoma.

## Funding

The authors received no specific funding for this work.

## Consent

We have obtained written consent from the patient’s parents.

## Conflicts of Interest

The authors declare no conflicts of interest.

## Data Availability

The data that support the findings of this study are available from the corresponding author upon reasonable request.
